# Supplementary material for: Cardiovascular Risk Factors and Their Association with Vitamin D Deficiency in Mexican Women of Reproductive Age
Source: Nutrients. 2019 May 28;11(6):1211. doi: 10.3390/nu11061211 (PMC6627884; doi:10.3390/nu11061211)
Supplement: Supplementary file 1 [file nutrients-11-01211-s001.pdf]

Supplementary Table 1. Multivariate logistic regression models for cardiovascular risk factors by Vitamin D Deficiency in Mexican women 20-49 years.

n sample=3,260		**Sedentarism** OR (CI 95%)	**Overweight** OR (CI 95%)	**Obesity** OR (CI 95%)	**IR** OR (CI 95%)	**T2DM** OR (CI 95%)	**HBP** OR (CI 95%)
VDD	No	1	1	1	1	1	1
	Yes	1.16 (0.83, 1.64)	1.32 (0.87, 2.0)	1.53 (1.02, 2.32)[a]	1.48 (1.04, 2.1)[a]	2.58 (1.65, 4.03)[a]	1.24 (0.84, 1.81)
Overweight+Obesity	No	1			1	1	1
	Yes	1.04 (0.75, 1.46)			2.27 (1.4, 3.69)[a]	3.73 (1.79, 7.78)[a]	2.08 (1.25, 3.49)[a]
Sedentarism	No		1	1	1	1	1
	Yes		1.17 (0.82, 1.68)	0.97 (0.64, 1.46)	1.13 (0.75, 1.71)	0.98 (0.59, 1.64)	1.10 (0.69, 1.74)
Age group	20 a 29 y	1	1	1	1	1	1
	30 a 39 y	1.4 (0.98, 1.99)[b]	2.64 (1.76, 3.95)[a]	4.52 (3.03, 6.73)[a]	1.22 (0.84, 1.78)	1.69 (0.82, 3.49)	0.83 (0.50, 1.39)
	40 a 49 y	1.53 (1.02, 2.29)[a]	3.21 (2.1, 4.92)[a]	5.09 (3.11, 8.31)[a]	1.11 (0.72, 1.71)	6.11 (2.98, 12.54)[a]	1.83 (1.08, 3.07)[a]
HBP	No	1	1	1	1	1	
	Yes	1.12 (0.7, 1.78)	1.3 (0.77, 2.21)	3.17 (1.73, 5.84)[a]	1.53 (1.04, 2.27)[a]	1.44 (0.81, 2.54)	
T2DM	No	1	1	1			1
	Yes	1.02 (0.6, 1.74)	2.33 (1.01, 5.39)[a]	3.71 (1.73, 7.95)[a]			1.37 (0.77, 2.44)
TG	<150 mg/dL	1	1	1	1	1	1
	>=150 mg/dL	0.89 (0.66, 1.21)	1.73 (1.21, 2.49)[a]	2.54 (1.75, 3.69)[a]	2.15 (1.5, 3.08)[a]	1.77 (1.03, 3.04)[a]	1.07 (0.74, 1.55)
HDL-C	>=50 mg/dL	1	1	1	1	1	1
	<50 mg/dL	0.95 (0.62, 1.47)	2.26 (1.48, 3.46)[a]	5 (3.23, 7.76)[a]	3.52 (1.98, 6.24)[a]	2.04 (0.91, 4.58)[b]	1.44 (0.89, 2.33)
TC	<200 mg/dL	1	1	1	1	1	1
	>=200 mg/dL	0.92 (0.66, 1.28)	1.07 (0.69, 1.65)	0.95 (0.63, 1.44)	0.63 (0.43, 0.93)[a]	0.97 (0.55, 1.74)	0.96 (0.67, 1.37)
Hcy	<10.4 umol/L	1	1	1	1	1	1
	>=10.4 umol/L	1.44 (0.54, 3.86)	0.47 (0.19, 1.21)	0.63 (0.24, 1.67)	1.12 (0.28, 4.62)	0.23 (0.05, 1.1)[b]	5.07 (2.41, 10.6)[a]
CRP	<5 g/L	1	1	1	1	1	1
	>=5 g/L	0.65 (0.47, 0.92)[a]	1.47 (0.86, 2.51)	4.29 (2.61, 7.07)[a]	2.7 (1.84, 3.95)[a]	1.54 (0.91, 2.59)	1.55 (1.05, 2.29)[a]
Area	Rural	1	1	1	1	1	1
	Urban	0.99 (0.74, 1.33)	1.06 (0.77, 1.45)	1.65 (1.15, 2.36)[a]	1.81 (1.26, 2.6)[a]	1.22 (0.73, 2.03)	0.94 (0.66, 1.34)
Region	South	1	1	1	1	1	1
	Center	0.91 (0.66, 1.27)	0.83 (0.57, 1.23)	0.87 (0.59, 1.29)	0.8 (0.49, 1.32)	0.59 (0.34, 1.05)[b]	1.40 (0.96, 2.05)[b]
	North	0.78 (0.57, 1.06)	0.83 (0.56, 1.24)	0.89 (0.58, 1.38)	0.91 (0.58, 1.44)	0.93 (0.53, 1.63)	1.40 (0.94, 2.01)[b]
SES	Tertile 1	1	1	1	1	1	1
	Tertile 2	0.66 (0.48, 0.9)[a]	1.26 (0.86, 1.86)	0.8 (0.52, 1.22)	0.8 (0.52, 1.23)	0.56 (0.3, 1.04)[b]	1.20 (0.78, 1.84)[b]
	Tertile 3	0.75 (0.52, 1.07)	1.10 (0.72, 1.68)	0.83 (0.53, 1.29)	0.75 (0.46, 1.23)	0.64 (0.36, 1.14)	0.81 (0.49, 1.32)
Indigenism	No	1	1	1	1	1	1
	Yes	1.17 (0.78, 1.77)	2.07 (1.31, 3.28)[a]	0.96 (0.58, 1.6)	0.92 (0.59, 1.43)	0.73 (0.35, 1.55)	0.63 (0.36, 1.11)

n sample=3,260		**HDL-C** OR (CI 95%)	**TC** OR (CI 95%)	**TG** OR (CI 95%)	**CRP** OR (CI 95%)	**Hcy** OR (CI 95%)	**AMI** OR (CI 95%)
VDD	No	1	1	1	1	1	1
	Yes	0.73 (0.48, 1.1)	1.45 (1.05, 2.01)[a]	1.27 (0.91, 1.77)	1.08 (0.75, 1.53)	1.01 (0.47, 2.15)	1.09 (0.48, 2.47)
Overweight+Obesity	No	1	1	1	1	1	1
	Yes	3.02 (2.12, 4.3)[a]	1.08 (0.74, 1.57)	2.03 (1.48, 2.79)[a]	2.57 (1.63, 4.06)[a]	0.63 (0.27, 1.46)	1.39 (0.52, 3.74)
Sedentarism	No	1	1	1	1	1	1
	Yes	0.97 (0.64, 1.47)	0.9 (0.65, 1.24)	0.88 (0.65, 1.2)	0.66 (0.47, 0.92)	1.41 (0.54, 3.7)	0.75 (0.29, 1.96)
Age group	20 a 29 y	1	1	1	1	1	1
	30 a 39 y	0.73 (0.45, 1.18)	1.71 (1.11, 2.62)[a]	1.37 (0.96, 1.96)[b]	1.17 (0.79, 1.74)	1.06 (0.4, 2.77)	1.98 (0.62, 6.41)
	40 a 49 y	0.69 (0.43, 1.1)	2.03 (1.39, 2.98)[a]	2.36 (1.66, 3.35)[a]	1.09 (0.72, 1.64)	2.23 (0.83, 5.98)	3.29 (0.9, 12.12)[b]
HBP	No	1	1	1	1	1	1
	Yes	1.45 (0.88, 2.39)	0.95 (0.67, 1.36)	1.11 (0.76, 1.61)	1.55 (1.04, 2.3)[a]	4.4 (2.28, 8.53)[a]	4.12 (1.87, 9.05)[a]
T2DM	No	1	1	1	1	1	1
	Yes	2.06 (0.86, 4.96)	0.92 (0.51, 1.66)	1.74 (1, 3.03)[b]	1.45 (0.85, 2.48)	0.29 (0.09, 0.93)[a]	1.23 (0.38, 4.01)
TG	<150 mg/dL	1	1		1	1	1
	>=150 mg/dL	3.94 (2.48, 6.25)[a]	4.94 (3.41, 7.17)[a]		1.14 (0.8, 1.61)	2.22 (0.83, 5.91)	0.91 (0.37, 2.23)
HDL-C	>=50 mg/dL		1	1	1	1	1
	<50 mg/dL		0.37 (0.23, 0.58)[a]	5.28 (3.31, 8.42)[a]	1.16 (0.74, 1.81)	1.24 (0.49, 3.12)	2.12 (0.48, 9.55)
TC	<200 mg/dL			1	1	1	1
	>=200 mg/dL			4.92 (3.4, 7.14)[a]	1.18 (0.84, 1.67)	2.49 (1.12, 5.55)[a]	0.78 (0.33, 1.83)
Hcy	<10.4 umol/L	1	1	1	1		
	>=10.4 umol/L	0.98 (0.39, 2.46)	2.66 (1.1, 6.45)[a]	2.16 (0.76, 6.19)	0.53 (0.18, 1.57)		
CRP	<5 g/L	1	1	1		1	1
	>=5 g/L	1.14 (0.73, 1.78)	1.19 (0.84, 1.67)	1.16 (0.82, 1.64)		0.56 (0.23, 1.37)	0.9 (0.38, 2.16)
Area	Rural	1	1	1	1	1	1
	Urban	0.72 (0.51, 1.01)[b]	1.11 (0.81, 1.51)	0.73 (0.52, 1.02)[b]	1.53 (1.1, 2.12)[a]	1.41 (0.68, 2.93)	3.75 (1.39, 10.12)[a]
Region	South	1	1	1	1	1	1
	Center	0.87 (0.57, 1.32)	0.9 (0.64, 1.28)	0.65 (0.46, 0.92)[a]	1.11 (0.79, 1.56)	1.09 (0.51, 2.33)	0.6 (0.26, 1.43)
	North	1.46 (0.93, 2.28)	1.46 (1.04, 2.04)[a]	0.41 (0.29, 0.58)[a]	1.55 (1.11, 2.18)[a]	0.53 (0.14, 2.05)	1.07 (0.38, 2.99)
SES	Tertile 1	1	1	1	1	1	1
	Tertile 2	1.04 (0.66, 1.65)	1.14 (0.78, 1.66)	1.04 (0.75, 1.43)	0.75 (0.54, 1.06)[b]	1.04 (0.49, 2.2)	0.43 (0.16, 1.13)[b]
	Tertile 3	0.74 (0.46, 1.2)	1.08 (0.72, 1.61)	0.89 (0.61, 1.28)	0.63 (0.43, 0.91)[a]	0.36 (0.12, 1.11)[b]	0.44 (0.16, 1.19)
Indigenism	No	1	1	1	1	1	1
	Yes	1.23 (0.67, 2.26)	0.59 (0.37, 0.95)[a]	0.98 (0.6, 1.59)	0.5 (0.31, 0.82)[a]	1.31 (0.52, 3.28)	0.39 (0.08, 1.94)

BMI: Body Mass Index, T2DM; Type 2 Diabetes Mellitus, IR; insulin resistance, HBP; high blood pressure, TC; total cholesterol, HDL-C; High density lipoprotein, TG; triglycerides, AMI; acute myocardial infarction, Hcy; homocysteine, CRP; C-reactive protein.
